# Supplementary material for: Noninvasive spinal stimulation safely enables upright posture in children with spinal cord injury
Source: Nat Commun. 2021 Oct 6;12:5850. doi: 10.1038/s41467-021-26026-z (PMC8494794; doi:10.1038/s41467-021-26026-z)
Supplement: Supplementary file 1 — Supplementary Information [file 41467_2021_26026_MOESM1_ESM.pdf]

Supplementary Information

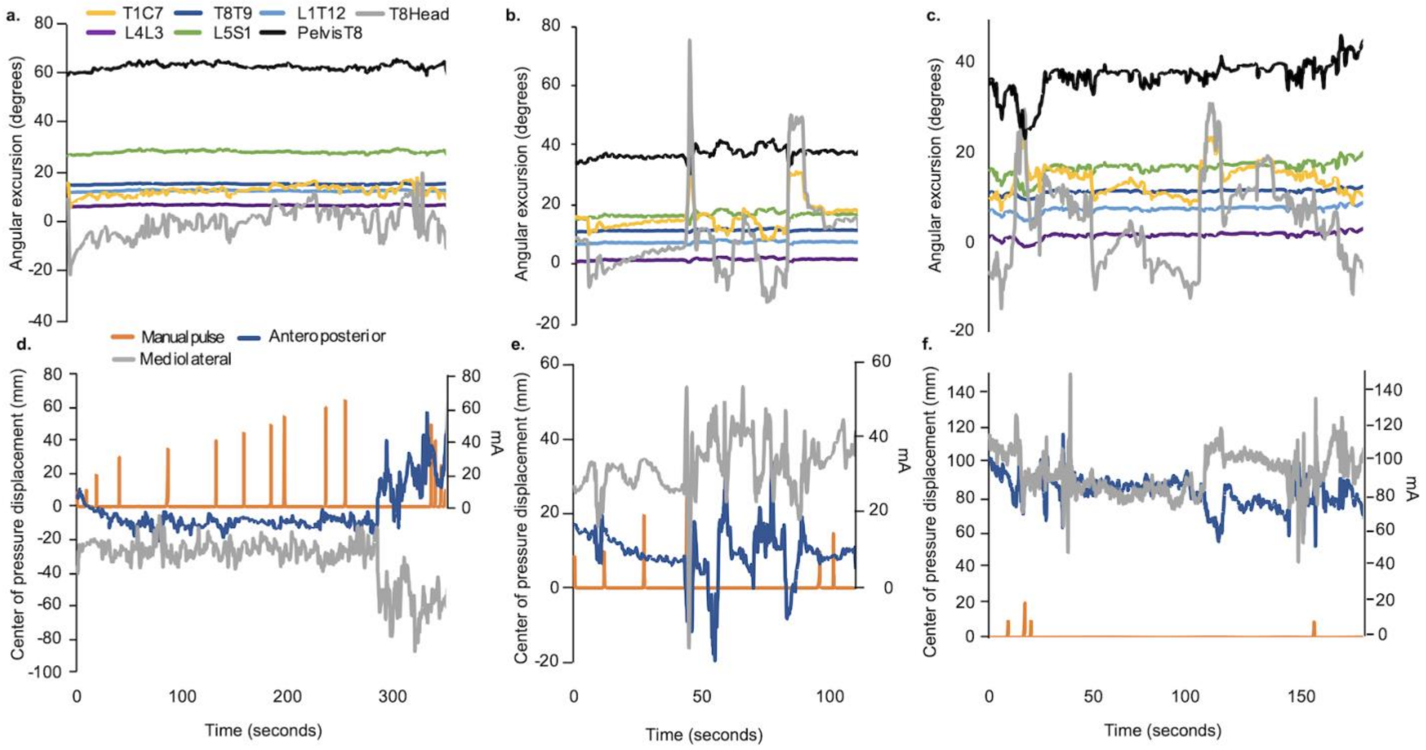

Supplementary Figure 1. Sitting posture during transcutaneous spinal cord (scTS) stimulation at C5 in children with SCI. Segmental trunk kinematics during scTS optimization for participants P14 (a), P5 (b), P34(c) for whom cervical stimulation was assessed on th third day. Anteroposterior and mediolateral center of pressure displacements (millimeters, (mm)) were recorded concomitant with kinematics P14 (d), P5 (e), P34 (f). Manual pulse indicates the increase of stimulation intensity in 10 milliamp (mA) increments. The rationale for including cervical stimulation specifically, comes from previous studies in healthy adults that demonstrated cervical stimulation to potentiate motor output in the lower limbs likely via amplification of residual descending drive and/or descending propriospinal system. However, in the current pilot study we did not see obvious changes in posture with cervical stimulation. Furthemore, cervical stimulation was above the level of injury for most of the tested participants. Some particiapnts reported heightened sensitivity in the region of cervical stimulation. Therefore the range of tested scTS intensities was relatively lower at C5 compared to those at T11 and L1 (20-65 mA, Table 1)

Supplementary Note 1. University of Louisville Institutional Review Board approved study protocol.

Transcutaneous spinal stimulation: Augmenting training for attaining intrinsic trunk control in children with spinal cord injury

Background

Damage to the descending pathways as a result of spinal cord injury (SCI) leads to the immediate dysfunction of multiple physiological systems below the level of injury (1). Like adults, children with SCI suffer from neuromuscular paralysis which results in the inability to sit upright, stand and walk (2). Current therapeutic interventions largely aim to compensate for paralysis assuming its permanence as a result of irreversible damage to the central nervous system. These interventions fail to prevent the unique secondary health complications following pediatric-onset SCI, i.e. scoliosis and hip dysplasia (3, 4). The discovery of the "intelligent" spinal cord that contains complex neuronal networks capable of generating rhythmic and coordinated motor patterns (5, 6), the central pattern generator (CPG) for locomotion, has set forth a major paradigm shift in our expectation of what is possible in terms of recovery after even the most severe SCI (7, 8). Studies have demonstrated, first, that after SCI, the CPG can be "accessed", reactivated and retrained via sensory feedback arising from the muscles and joints during activity-based locomotor training (AB-LT) (9-14). Second, application of epidural and transcutaneous stimulation (TcStim) to the spinal cord below the level of lesion can augment the neuromuscular capacity for voluntary movement, standing and stepping in individuals with chronic motor complete SCI (8, 15-17). Researchers propose that neuromodulation raises the central state of excitability of the neuronal networks enabling greater capacity to integrate sensory input and produce sufficient motor output as a physiological mechanism for these improvements (8). Children with SCI may not only benefit from novel therapeutic approaches but also demonstrate even greater improvements in neuromuscular recovery due to inherent plasticity present in the stages of rapid growth and development. This is readily evident from our recent study in which 21 children had unexpected significant gains in intrinsic trunk control following AB-LT (18). Some children attained full trunk control, while others plateaued prior to reaching full recovery. Recently, TcStim was shown to acutely improve sitting posture and trunk muscle activation in adults with SCI, (19) while in children with cerebral palsy, a combination of AB-LT with TcStim intervention resulted in greater improvement of locomotion as compared to AB-LT alone (20). The evidence from these studies, published minimal risk for skin redness, and the non-invasive nature of TcStim (i.e. stimulation through electrode on the skin) make it a potentially feasible and attractive technique to apply with AB-LT to further facilitate neuromuscular recovery in children with SCI. Our overall objective is to test 'proof-of-principle' that TcStim is a feasible and safe approach to improve sitting posture/trunk control via increased trunk muscle activation below the lesion in children with SCI and impaired trunk control.

A. Objectives

Specific Aim 1: Determine a) proof-of-principle and b) feasibility and safety of TcStim for acute potentiation of the upright sitting posture and trunk control in children with SCI.

Hypothesis 1: TcStim is a safe and feasible approach to acutely potentiate upright sitting posture and trunk control in children with SCI.

Specific Aim 2: Determine the feasibility and safety of TcStim in combination with activity-based therapy for potentiating upright posture and trunk control in children with SCI.

Hypothesis 2: TcStim in combination with 40 AB-LT training sessions is a safe and feasible approach to potentiate upright sitting posture and trunk control in children with SCI.

The current study serves as a foundational step providing necessary preliminary data to advance the study of TcStim as a neurotherapeutic agent from adults with SCI to the pediatric population with SCI. Completion of the proposed aims will test and establish safety and feasibility, including risk likelihood for use of TcStim neuromodulation alone and in combination with AB-LT to improve upright sitting posture and trunk control in children with SCI. Furthermore, the results will provide initial proof-of-principle of therapeutic potential for

TcStim alone to acutely improve sitting posture and trunk control. This data will be used in future grant applications aimed to investigate the efficacy of TcStim for recovery of upright sitting posture and trunk control in children with SCI.

B. Methodology

Sample: For Aim 1: Ten participants will be enrolled, ages 2-15 years who meet the following inclusion criteria: history of chronic, acquired upper motor neuron SCI (traumatic or non-traumatic); discharged from in-patient rehabilitation, trunk control deficit as either documented with the Segmental Assessment of Trunk Control (SATCo, score < 20) or reported/observed inability to sit fully upright and without use of arm support or difficulty reaching while maintaining posture. Exclusion criteria include botox use within past 3 months; current baclofen use, unhealed fracture, any other medical complication limiting participation in the assessments; prior surgery for scoliosis; congenital SCI and total ventilator dependence.

For Aim 2: Four participants will be enrolled, age 2-15 years who meet the following inclusion criteria: history of chronic, acquired upper motor neuron SCI (traumatic or non-traumatic); discharged from in-patient rehabilitation, trunk control deficit as either documented with the Segmental Assessment of Trunk Control (SATCo, score < 20) or reported/observed inability to sit fully upright and without use of arm support or difficulty reaching while maintaining posture, and history of completion of a minimum of 60 sessions of activity-based locomotor training/therapy at Frazier Rehab. Exclusion criteria includes: botox use within past 3 months; current baclofen use, unhealed fracture, any other medical complication limiting participation in the assessments and/or activity-based locomotor training; prior surgery for scoliosis; congenital SCI and total ventilator dependence. Participants enrolled in Aim 1 may also participate in Aim 2 of the study, if they meet the eligibility criteria for Aim 2. The study physician screens all candidates for participation.

Figure 1. Study Design

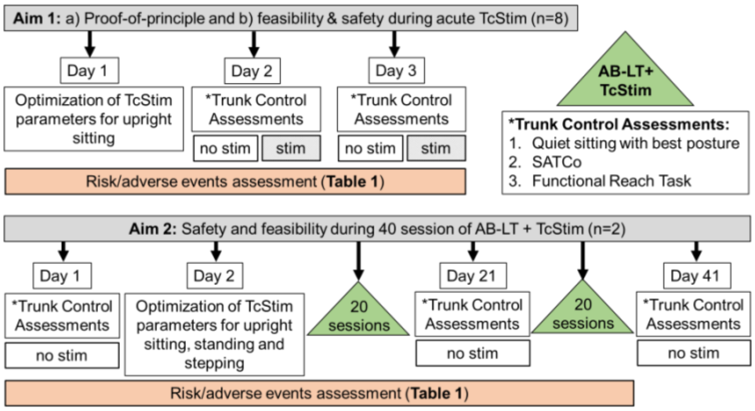

Study Design (Figure 1): This is a within subjects, repeated measures study design. For Aim 1a, proof-of-principle will be determined by a significant and meaningful, acute change in trunk control measured by trunk angular excursions (flexion/extension), center of pressure (COP) displacement, trunk muscle electromyography (EMG) and SATCo score – collected over two conditions (no stim/stim). For Aim 1b, safety and feasibility will be determined by the frequency count of anticipated and unanticipated risks and calculation of risk likelihood (See Table 1). For Aim 2, safety and feasibility will be determined by the frequency count of anticipated (Table 1) and unanticipated risks assessed over approximately 40 AB-LT + TcStim therapy sessions with the subsequent calculation of risk likelihood. We also will explore the impact of combined AB-LT + TcStim therapy on trunk control by comparing the outcomes at baseline, approximately post-20 and approximately 40 AB-LT sessions. Demographics related to etiology, age at the onset of SCI, time since injury will be collected according to NINDS-CDE guidelines for pediatric SCI (21). Transcutaneous spinal stimulation (TcStim): The 5-channel stimulator (22) generates pain-free biphasic rectangular waveform of 0.3- to 1.0-ms pulses with a frequency of 10 kHz. As reported in adults, intensity and frequency between 30-200 mA and 5 to 40 Hz will be used, respectively [8]. Participant preparation (Aim 1 & 2): The participants will be instructed to empty the

Table 1. Safety and feasibility measures.

Measures	Pre-stim	Stim-on	End of session
Skin (irritation, redness)	X		X
Vital signs (BP/HR)	X	X	X
Autonomic dysreflexia		X	X
Bowel accident		X	
Pain	X	X	X
Numbness/Tingling	X	X	X
Fatigue		X	X
Spasticity	X	X	X
Request to stop stim		X	
Regression of trunk function		X	X
Other risks (unknown)		X	X
Compliance (attendance)			X

bladder prior to arrival to the laboratory. The multi-muscle wireless EMG electrodes (Cometa) and motion trackers (X-sens) for kinematics will be placed on the participant's skin surface (see example of typically developing child in Figure 2). For TcStim, 2-3round electrodes (Syrtenty®, China) will be placed midline between cervical and low thoracic/high lumbar regions spinous processes, as cathodes and rectangular pads (Syrtenty®) placed symmetrically on the skin over the iliac crests and/or clavicle as anodes. At the beginning of each experiment the participant's skin will be examined for any redness or irritation, particularly in the areas of electrode placement. Throughout the study, the comfort and status of the participants will be carefully monitored. To monitor vital signs, blood pressure (BP) and heart rate (HR) will be measured prior and during experiment using continuous beat-by-beat BP (Finapres) and electrocardiography (ECG) (AD instruments) recording. If at any point during the TcStim, the participant presents with signs and symptoms of autonomic dysreflexia (AD), with characteristic rapid increase in BP and decrease in HR (23), the stimulation will be stopped, resolution of AD addressed immediately, and documented. Penn Spasm Scale (24) will be used to assess the incidence of spasms. FACES (3-8) and Visual Analog Scale (8+) (25, 26) will be presented during the experiment. The stimulation will be stopped at any point per participant's request or an observed negative response. The investigator will assess the event, cause, and risk to achieve resolution (Table 1). The total duration (dose) of TcStim during each training session will be recorded. Incidence of all anticipated risks/events and unanticipated risks/events will be documented. Participant compliance, i.e. regular attendance of the training sessions and follow-up experiments, will contribute to the assessment of feasibility of TcStim + AB-LT as a neurotherapy. Participants in both aim 1 and aim 2 may have video and images recorded.

The following will be covered in the consent process:

a. An explanation of the consent document in its entirety
b. Ensuring the participant has read the consent document
c. A review of each section of the consent form by stating its title
d. An explanation of all risks related to study participation
e. Confirmation the participant understands the study by asking questions
f. Obtaining the informed consent and confirming the appropriate signatures

A copy of the completed informed consent/assent document(s) will be provided to the participant. The consent document will be given to the Lead Regulatory Coordinator for processing.

C. Subject Recruitment Methods

Ten to fourteen participants will be enrolled in this study; ten participants for Aim 1 and four participants for Aim 2 Participants may take part in Aim 1 and Aim 2 of the study if eligible for both study aims. Recruitment will occur sequentially for Aim 1, then Aim 2.

The research team will utilize the Human Locomotion Research Center's Potential Volunteer Database (UofL Study #06.0647) to identify potential research volunteers based on eligibility criteria. Potential candidates will be informed of the study and its purpose and if interested, the study informed consent will be reviewed and questions answered by the PI. Potential candidates will be medically screened for eligibility by the study physician. Candidates willing to participate will sign consent (parent/guardian) with assent by children ≥7 years.

D. Informed Consent Process/complete HIPAA Waiver

This protocol will comply with the KSCIRC Standard Operating procedure for consent documentation and processing for research participants. The consent documents will be provided to the study participant (and Legally Authorized Representative [LAR], if applicable) up to 3 days prior to a consent meeting for their review by the PI or a member of the research team. An Assent will also be provided for all minors participating in the study who are at least 7 years of age. The PI or another qualified member of the research team will consent/assent the research participant and LAR prior to onset of any study procedure.

E. Experimental Protocol

Aim 1 (n=10), the data collection will occur over 3 separate days (Fig 1). Day 1 (TcStim optimization), after preparation (Fig. 2) the participant will be seated on the force plate (Burtec, FP4060-NC-1000). Trunk kinematics, EMG, and sitting COP (**Figure 3**) data will be recorded at baseline during 3 attempts of best upright sitting

Figure 3. Physiological Outcome Measures for Assessment of Trunk

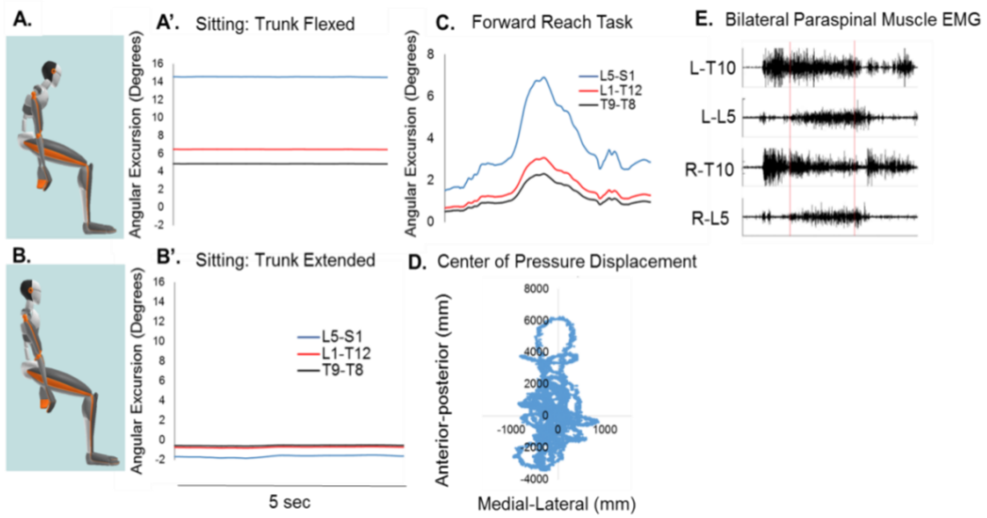

Figure 3. Physiological outcome measures. Wireless motion tracker system creates a 3D avatar and provides real-time interactive visual feedback of the body position (**A&B**). Sitting with 'C' posture (trunk flexed) results in greater flexion at 3 trunk segments (**A'**) as compared to sitting with upright posture (trunk extended) (**B'**). Change in angular excursion (**C**) during seated forward reach on force plate is accompanied by anterior shift in Center of Pressure displacement (**D**). Paraspinal muscles EMG in a typically developing child during seated trunk extension (L- left, R-right) (**E**)

posture. TcStim will be turned on and intensity, frequency, and electrode placement will be modulated to obtain the upright sitting posture, indicated by trunk extension (**Fig.3 A→ B**) with concomitant COP displacement. The optimal stimulation parameters during the 3 sitting upright attempts with TcStim will be noted. Day 2 and Day 3: Data collection during trunk control assessments: 1) quiet sitting with best upright sitting posture 2) SATCo test and 3) functional reach test will be performed in two experimental conditions (randomized): no stim and TcStim with optimized parameters for upright posture. The participant response will be monitored, as described (**Table 1**). In case of any unanticipated circumstances during Day 2 (e.g. event that results in incomplete data collection), an additional day will be used to collect the data (Day 3) to ensure the acquisition of the full data set. **Aim 2 (n=4)**: The participants will undergo AB-LT for 1.5 hours/day 5x/week for approximately 40 sessions in combination with TcStim. Day 1: Baseline data collection during trunk control assessments. Day 2: TcStim optimization for upright sitting, standing and stepping. AB-LT + TcStim Intervention: Children will be fitted with a trunk-pelvic harness for AB-LT. AB-LT sessions will occur daily (5x/week) for approximately 40 sessions and consist of approximately 55–60 min on the treadmill (TM) for facilitated standing/stepping followed by approximately 30 min of activities off the TM (over ground). AB-LT provides task-specific sensory input reinforcing upright, appropriate alignment of the trunk, pelvis and lower extremities during weight bearing stepping with proper inter/intra-limb coordination including arm swing (13, 14, 18). Throughout the AB-LT session pediatric physical therapists use play oriented activities to promote active trunk extension and rotation. These activities may include placing balls or toys overhead or to the child's side for reach and grasp, shooting baskets or carefully removing pieces of a puzzle tower while maintaining a stable trunk. Sixty minutes on the TM are divided into ~ 5-10 minute bouts of stepping and standing with TcStim administered intermittently for duration of ~ 5 min and no more than 10 minutes at a time (total of ~6X5min of TcStim). The TM speed and amount of body weight support (BWS) may be adjusted based on the participant's response. The training parameters (time, speed, %BWS) will be recorded for each session. TcStim will be delivered intermittently (5 – 10 min bouts) during the over ground training activities in sitting, standing and stepping; stimulation parameters will be recorded. Day 21 (post approximately 20 AB-LT + TcStim) and Day 41 (post approximately 40 AB-LT +TcStim): Trunk control assessments will be performed for exploratory analysis.

F. Potential Risks.

The primary goal of this study is to determine the safety and feasibility of transcutaneous stimulation. Based on the previous studies investigating this technique in adults with spinal cord injury (27, 28) and children with cerebral palsy (ages 6-11) (20) there were no serious adverse effects associated with the stimulation. Mild and painless hyperemia of the skin (redness) under the stimulation electrode was reported as the only occurring risk and this dissipates within 5-10 min (28). We will be assessing and monitoring the participant continuously throughout the study for any of the following possible risks that may occur during participation in the study. The risks are divided according to those that may occur during the stimulation protocol and those that may occur during activity-based locomotor training.

Risks of stimulation:
Likely:

- Skin irritation from the stimulation or electrodes
- Mild changes in blood pressure and heart rate
- Pain
- Tingling or tickling feeling
- Spasticity

Less Likely:

- Autonomic dysreflexia
- Bowel accident
- Fatigue, become tired
- Numb feeling from stimulation
- Headache or neck ache
- Undesirable change in stimulation

Rare:

- Regression of trunk control

Risks of activity-based locomotor training:
Likely:

- Skin irritation from the harness and trainers' manual cues
- Muscle soreness

Less Likely:

- Fatigue, become tired

Rare:

- Fall
- Joint sprain
- Broken bone

Unless a participant is injured, there is no financial risk to the participant resulting from participation in this research protocol. If a participant is injured by being in this research study, the study doctor will arrange for the participant to receive medical treatment. There is no money set aside to pay for treatment of any injury. The participant's insurance will be billed for the treatment of these injuries. Before the participant agrees to take part in the research study s/he should find out if insurance will cover an injury in this kind of research. There is no money set aside for lost wages, discomfort, disability, etc. In the event of a research related injury, the participant will be advised to call Dr. Jennifer Thompson at 502-588-3440 or the Center for Pediatric NeuroRecovery at 502-569-7996.

Minimizing Risk

Participant Confidentiality

To protect confidentiality, each research participant will be assigned a coded identifier with no association to their identity. This identifier will distinguish all evaluations and analyses. Data will be stored on computer media and video and will be secured in a locked storage area. Only members of the research team including research staff, post-doctoral associates and graduate students will have access to the data for analyses. The PI will have access to the coding of the coded identifier to the research participants.

Minimizing Procedural Risks

The PI for this project is an experienced physical therapist with clinical expertise in SCI, both adults and pediatric, use of the body weight support system and harness and provision of locomotor training to the pediatric population, and movement/physiologic research in children with spinal cord injury. A study co-investigator has expertise in the use of neuromodulation/stimulation techniques. Research post-doctoral fellows are trained in the acquisition of EMG, motion, force data and monitoring of BP/heart rate in adults and children and in the use of stimulation. A pediatric physical or occupational therapist will be present for all experiments and training sessions and leads the delivery of activity-based therapy as well as advocacy/monitoring of the participant throughout each experiment and training session. The research assistants are trained staff in locomotor training and safety procedures for adults and children. Each will use their expertise to provide a safe and comfortable testing and training environment.

At the beginning of each experiment the participant's skin will be examined for any redness or irritation, particularly in the areas of electrode placement. To monitor vital signs, blood pressure (BP) and heart rate (HR) will be measured prior and during experiment using continuous beat-by-beat BP (Finapres) and electrocardiography (ECG) (AD instruments) recording. A history of autonomic dysreflexia will be reviewed with the parent and child to see if this participant has any recent experience of autonomic dysreflexia. The child will be asked to empty the bladder prior to locomotor training or testing. If at any point during the TcStim, the participant presents with signs and symptoms of autonomic dysreflexia (AD), with characteristic rapid increase in BP and decrease in HR (23), the stimulation will be stopped, resolution of AD addressed immediately, and documented. Throughout the study, the participant will be carefully monitored. The transcutaneous stimulator that will be used in the current study uses a stimulation waveform that does not elicit pain when used at the intensities required to transcutaneously (e.g. through the skin) activate the spinal networks (15). We will monitor the comfort and status of the participants throughout the study. Specifically, we will present FACES (3-8) and Visual Analog Scale (8+) (25, 26) during the experiment. Penn Spasm Scale (24) will be used to assess the incidence of spasms. The stimulation will be stopped at any point per participant's request or an observed negative response. The investigator will assess the event, cause, and risk to achieve resolution (**Table 1)**. The total duration (dose) of TcStim during each training session will be recorded. Incidence of all anticipated risks/events and unanticipated risks/events will be documented.

The discomfort and health risks for participants participating in this study are minimal. The same precautions and safety guidelines will be taken that are provided in patient care in rehabilitation settings. To reduce the risk of falling during standing and stepping, the participant will wear a support harness attached to a body weight support system that will provide support and act as a safety catch if the participant should stumble or fatigue on the treadmill. The harness will be fit for each participant and re-adjusted should the participant report any

discomfort. The patient's skin is checked before and after training for any redness. The participant will be oriented to the equipment and the safety catch to assure its effectiveness. In this study, participants will already have experience with the locomotor training environment and therapy having previously received a minimum of 60 therapy sessions.

The participant will be introduced and familiarized to all testing environments. Each participant's experiment/study readiness will be evaluated on an on-going basis by the Research Physical Therapist/Occupational Therapist. Rest periods will be incorporated into the training and/or testing procedures. The participant will be allowed to rest at any point, as requested.

Participants will be continuously monitored for any signs of discomfort or risks by a pediatric physical therapist, an activity-based technician or an occupational therapist before, during and after every assessment and intervention session. The criteria for monitoring and reviewing the child's condition is defined by each test's risks and intervention risks. The research team member (therapist or technician) observes and documents the participant's study readiness, responses during the course of the study, and outcomes including any signs of discomfort or occurrence of a potential risk. If discomfort or any risks occur, the session will be immediately halted and the therapist evaluate and attempt, as appropriate, to resolve either the discomfort and/or the risk potential. The therapist may also terminate the experiment due to the discomfort or risk. If the study is terminated, the Principal Investigator is contacted immediately for follow up. If needed, the study physician will be notified and the individual referred to the study physician. Immediate medical care will be provided when necessary. If an event has occurred that does not require medical care (e.g. skin irritation), a research staff member contacts the parent/caregiver to follow-up on the participant's status to resolution. The participant's status prior, during, and upon completion of the study is documented by a research team member. A list of events or lack thereof is provided weekly to the study physician for review and on-going risk assessment. The PI also reviews this list weekly to assess and document adverse events relative to the individual and on-going study risk assessment.

G. Plan for Analysis.

Aim 1: As a proof-of-principle, it is anticipated that TcStim will result in an immediate and significant (clinically meaningful) change in sitting posture quantified by angular excursion of trunk segments, center of pressure displacement, and EMG activation during trunk control assessments. (Fig.3) A within subjects repeated measures design with p value of 0.05 level of significance will be used to compare the physiological measures between no-stim and stim conditions for each participant within the group. The current study protocol, including the sample size of 8 participants, is based on the published study assessing the effects of TcStim on trunk control in adults with SCI (29) A sample size of 8 provides 80% power to detect an effect size of 1.2 (classified as very large on the Cohen's scale (30) extended by Sawilowsky (31)) on a pre-post continuous measure using 2-sided paired t-test with a significance level of 0.05. Rath et al(29) found that the no-stim to stim angular excursion changes were 4.74±5.41mm to 1.36±0.98 mm. Assuming a moderate pre-post correlation of 0.5, the no-stim to stim standard deviation corresponding to that change is 4.99 (using: $SD_{pre-post} = \sqrt{SD_{pre}^2 + SD_{post}^2 - 2Corr_{pre-post}SD_1SD_2}$). If assuming a similar angular excursion, for this study, this effect size of 1.2 corresponds to a no-stim to stim angular excursion decrease of 5.99mm. This sample size of 8 will be adequate to obtain the angular excursion variabilities in children which will help in the statistical planning of a larger study, sufficiently powered to detect a smaller effect size. We have designated an n=10 to allow for any drop-outs or study incompletions.

Aim 2: To evaluate the safety and feasibility of TcStim application in children with SCI, the risk likelihood of adverse events, anticipated (listed in Table 1) and unanticipated risks will be documented for n of 4 who will undergo repetitive and cumulative exposure to TcStim (approximately 40 sessions). Adverse events in 'real-time' will also be addressed to discern level of risk and ability to continue experiment/study and the need toalter our approach. The risk likelihood will be presented in terms of percentage of occurrences. This information will be relevant to future grant applications to establish known risks and risk-benefit for TcStim protocols and informed consent specifically for children and adolescents with SCI.

We will also present the likelihood of study completion as a percentage of the number of sessions completed compared to the number of planned sessions. Our current clinical compliance rate is 91% for out-patient therapy sessions attended by 40 pediatric patients with absences for illness, travel difficulties, inclement weather, or family issues. Our current compliance for children/adolescents participation in research studies is 100% completion for > 100 visits. We have had to re-schedule occasionally, but all participants have completed each study session indicating logistic feasibility for full participation by children age 18 months to 18 years. Experiment sessions are kept to under 2 hours total and includes: documentation of examination of the participant for study readiness, preparation of the participant, conducting the experiment, removal of electrodes, tape, etc., and examination/documentation of participant readiness for departure. In some instances, we are cognizant of an age limit for participation, e.g. inability to follow instructions for respiratory function tests for children under age 4-5 years; inability to voice, understand or describe possible discomfort/pain. There may possibly be an age/cognitive limitation for use of TcStim in pediatrics and we will be assessing across ages to understand any such limits. A compliance rate comparable to the clinic (>90%) would be appropriate for the combined AB-LT and TcStim, whereas >95% compliance rate for experiments is expected allowing for any incidents preventing experiment completion on one day, but allowable on another.

Only an exploratory analysis will be conducted to examine the effect of AB-LT and TcStim combined on trunk control assessments. We will not base future studies on this outcome, but instead on the proof-of-principle outcome (Aim 1) as we did not select a statistical ‘n’ to examine the effect of long-term combined therapy on trunk control. The secondary data analyses include obtaining variabilities (change in standard deviations, within-individual variability, e.g.) needed for planning of a future larger study. In order to obtain these variabilities, the data obtained will be analyzed with generalized mixed models, which will include children’s characteristics as fixed factors, random intercept to account for in-person variability and random slope of time to account for individual pattern over time. These models will be appropriate for the longitudinal nature of the data. We will be interested in recording the model intercept estimate and the standard errors of baseline, slope of time and residual for each outcome. Our intent in this current project is not hypothesis testing but rather obtaining estimates needed for a future sample size calculation. Therefore, we will not be interested in statistical significance of any of our estimates. This statistical work will be performed in SAS 9.4 (SAS Inc, Cary, NC).

I. Research Materials, Records, and Privacy

Adequacy of Protection Against Risk

Recruitment of participants may be performed through our secure research database that includes over 5,000 individuals registered with SCI. In addition, Frazier Rehab Hospital serves individuals with SCI who may be referred to our research program. All potentially eligible research participants will meet at Frazier Rehab Institute to discuss the complete protocol, including risks and benefits with Principle Investigator and/or designated research staff. The informed consents will be written in lay language and will contain information on all procedures to be performed as well as contact information if the research participant and his/her associates should have any questions. All potential research participants will be encouraged to read the informed consent and discuss the study with their physician, family and friends, before signing. The original signed informed consents will be kept in a locked cabinet in a locked room within an area of controlled access. A scanned copy will be stored on our server with password protection. Eligibility checklists will be signed by Principal Investigator with all source documentation and stored in a locked cabinet in a locked room within an area of controlled access. A scanned copy will be stored on our server with password protection.

If a participant is a child of someone employed by the University of Louisville, we will review and discuss a risk management plan. The plan will be signed by the investigator and the participant and witnessed by an individual outside of the study and department to ensure there is no coercion. A copy of the plan will be provided to the participant and placed in his/her employee file.

Security

Any paper documents containing information that identifies participants (including informed consent documents) will be stored in a locked office and, whenever possible, within a locked cabinet. Any electronic files containing identifying information will be stored on university-secured computers. When travel is necessary, laptops containing files with identifying information are password protected, and when accessing documents, files are further secured via the University of Louisville's VPN (virtual private network).

Risks of confidentiality breach

To protect confidentiality, each research participant will be assigned a Kentucky Spinal Cord Injury Research Center (KSCIRC) research participant ID number. This becomes the participant's coded identifier with no association to their identity. This identifier will distinguish all evaluations and analyses. All data will be stored on computer media and video and will be password protected, all written data will be secured in a locked storage area. Only members of the research team including research staff, post-doctoral students and graduate students will have access to the data for analyses. The Principal Investigator will have access to the coding of the coded identifier to the research participants.

In order to publish this data, another publication identification (Pub ID) number will be assigned. The master list for this number is in a database with restricted, password-protected access by appropriate members of the study team.

Literature Cited

1. Sweis R, Biller J. Systemic Complications of Spinal Cord Injury. Current neurology and neuroscience reports. 2017;17(2):8. Epub 2017/02/12. doi: 10.1007/s11910-017-0715-4. PubMed PMID: 28188542.
2. Vogel LC, Zebracki K, Betz R and Mulcahey MJ. Spinal cord injury in the child and young adult. London: Mac Keth Press 2014.
3. Mulcahey MJ, Gaughan JP, Betz RR, Samdani AF, Barakat N, Hunter LN. Neuromuscular scoliosis in children with spinal cord injury. Topics in spinal cord injury rehabilitation. 2013;19(2):96-103. Epub 2013/05/15. doi: 10.1310/sci1902-96. PubMed PMID: 23671379; PMCID: Pmc3641911.
4. Mehta S, Betz R, Mulcahey MJ, McDonald C, Vogel L. Effect of Bracing on Paralytic Scoliosis Secondary to Spinal Cord Injury. The journal of spinal cord medicine. 2004;27(sup1):S88-S92. doi: 10.1080/10790268.2004.11753448.
5. Brown GT. The Intrinsic Factors in the Act of Progression in the Mammal. Proceedings of the Royal Society of London. 1911;84(574):308-19.
6. Dimitrijevic MR, Gerasimenko Y, Pinter MM. Evidence for a spinal central pattern generator in humans. Annals of the New York Academy of Sciences. 1998;860:360-76. Epub 1999/02/03. PubMed PMID: 9928325.
7. Angeli CA, Boakye M, Morton RA, Vogt J, Benton K, Chen Y, Ferreira CK, Harkema SJ. Recovery of Over-Ground Walking after Chronic Motor Complete Spinal Cord Injury. The New England journal of medicine. 2018;379(13):1244-50. Epub 2018/09/25. doi: 10.1056/NEJMoa1803588. PubMed PMID: 30247091.
8. Angeli CA, Edgerton VR, Gerasimenko YP, Harkema SJ. Altering spinal cord excitability enables voluntary movements after chronic complete paralysis in humans. Brain : a journal of neurology. 2014;137(Pt 5):1394-409. Epub 2014/04/10. doi: 10.1093/brain/awu038. PubMed PMID: 24713270; PMCID: PMC3999714.
9. Behrman AL, Bowden MG, Nair PM. Neuroplasticity after spinal cord injury and training: an emerging paradigm shift in rehabilitation and walking recovery. Physical therapy. 2006;86(10):1406-25. Epub 2006/10/03. doi: 10.2522/ptj.20050212. PubMed PMID: 17012645.
10. Harkema SJ, Hurley SL, Patel UK, Requejo PS, Dobkin BH, Edgerton VR. Human lumbosacral spinal cord interprets loading during stepping. Journal of neurophysiology. 1997;77(2):797-811. Epub 1997/02/01. PubMed PMID: 9065851.
11. Dietz V, Muller R, Colombo G. Locomotor activity in spinal man: significance of afferent input from joint and load receptors. Brain : a journal of neurology. 2002;125(Pt 12):2626-34. Epub 2002/11/14. PubMed PMID: 12429590.
12. Behrman AL, Harkema SJ. Locomotor training after human spinal cord injury: a series of case studies. Physical therapy. 2000;80(7):688-700. Epub 2000/06/27. PubMed PMID: 10869131.
13. Harkema S, Behrman A, Barbeau H. Evidence-based therapy for recovery of function after spinal cord injury. Handb Clin Neurol. 2012;109:259-74. doi: 10.1016/B978-0-444-52137-8.00016-4. PubMed PMID: 23098718.
14. Behrman AL, Nair PM, Bowden MG, Dauser RC, Herget BR, Martin JB, Phadke CP, Reier PJ, Senesac CR, Thompson FJ, Howland DR. Locomotor training restores walking in a nonambulatory child with chronic, severe, incomplete cervical spinal cord injury. Physical therapy. 2008;88(5):580-90. doi: 10.2522/ptj.20070315. PubMed PMID: 18326054; PMCID: PMC2390720.
15. Gerasimenko Y, Gorodnichev R, Moshonkina T, Sayenko D, Gad P, Reggie Edgerton V. Transcutaneous electrical spinal-cord stimulation in humans. Annals of physical and rehabilitation medicine. 2015;58(4):225-31. Epub 2015/07/25. doi: 10.1016/j.rehab.2015.05.003. PubMed PMID: 26205686; PMCID: PMC5021439.
16. Mayr W, Krenn M, Dimitrijevic MR. Epidural and transcutaneous spinal electrical stimulation for restoration of movement after incomplete and complete spinal cord injury. Current opinion in neurology. 2016;29(6):721-6. Epub 2016/11/03. doi: 10.1097/wco.0000000000000382. PubMed PMID: 27798422.
17. Gorodnichev RM, Pivovarova EA, Pukhov A, Moiseev SA, Savokhin AA, Moshonkina TR, Shcherbakova NA, Kilimnik VA, Selionov VA, Kozlovskaia IB, Edgerton VR, Gerasimenko Iu P. [Transcutaneous electrical stimulation of the spinal cord: non-invasive tool for activation of locomotor circuitry in human]. Fiziologiia cheloveka. 2012;38(2):46-56. Epub 2012/06/12. PubMed PMID: 22679796.
18. Argetsinger LC, Trimble SA, Roberts MT, Thompson JE, Ugiliweneza B, Behrman AL. Sensitivity to change and responsiveness of the Segmental Assessment of Trunk Control (SATCo) in children with spinal cord injury. Developmental neurorehabilitation. 2018:1-12. Epub 2018/05/23. doi: 10.1080/17518423.2018.1475429. PubMed PMID: 29787329.

19. Rath M, Vette AH, Ramasubramaniam S, Li K, Burdick J, Edgerton VR, Gerasimenko YP, Sayenko DG. Trunk Stability Enabled by Noninvasive Spinal Electrical Stimulation after Spinal Cord Injury. Journal of neurotrauma. 2018;35(21):2540-53. Epub 2018/05/23. doi: 10.1089/neu.2017.5584. PubMed PMID: 29786465; PMCID: PMC6205803.
20. Solopova IA, Sukhotina IA, Zhvansky DS, Ikoeva GA, Vissarionov SV, Baindurashvili AG, Edgerton VR, Gerasimenko YP, Moshonkina TR. Effects of spinal cord stimulation on motor functions in children with cerebral palsy. Neuroscience letters. 2017;639:192-8. Epub 2017/01/09. doi: 10.1016/j.neulet.2017.01.003. PubMed PMID: 28063935.
21. Mulcahey MJ, Vogel LC, Sheikh M, Arango-Lasprilla JC, Augutis M, Garner E, Hagen EM, Jakeman LB, Kelly E, Martin R, Odenkirchen J, Scheel-Sailer A, Schottler J, Taylor H, Thielen CC, Zebracki K. Recommendations for the National Institute for Neurologic Disorders and Stroke spinal cord injury common data elements for children and youth with SCI. Spinal cord. 2017;55(4):331-40. Epub 2016/11/16. doi: 10.1038/sc.2016.139. PubMed PMID: 27845358.
22. A. Grishin A, Moshonkina T, Solopova I, M. Gorodnichev R, Gerasimenko Y. A Five-Channel Noninvasive Electrical Stimulator of the Spinal Cord for Rehabilitation of Patients with Severe Motor Disorders2017.
23. Eldahan KC, Rabchevsky AG. Autonomic dysreflexia after spinal cord injury: Systemic pathophysiology and methods of management. Autonomic neuroscience : basic & clinical. 2018;209:59-70. Epub 2017/05/17. doi: 10.1016/j.autneu.2017.05.002. PubMed PMID: 28506502; PMCID: PMC5677594.
24. Mills PB, Vakil AP, Phillips C, Kei L, Kwon BK. Intra-rater and inter-rater reliability of the Penn Spasm Frequency Scale in People with chronic traumatic spinal cord injury. Spinal cord. 2018;56(6):569-74. Epub 2018/01/27. doi: 10.1038/s41393-018-0063-5. PubMed PMID: 29371700.
25. Gilbert G, D'Souza, P., Pletz, B. Pain assessment and management-pediatric. San Mateo County EMS Agency.Pediatric treatment protocols.
26. Wong DL H-EM, Wilson D, Winkelstein ML. Wong’s Essentials of Pediatric Nursing. 6th ed. St. Louis: Mosby; 2001.
27. Rath M, Vette, AH., Ramasubramaniam, S., Li.K., Burdick,J., Edgerton, V. R., Gerasimenko, Y.P., Sayenko, D.G. Trunk Stability Enabled by Noninvasive Spinal Electrical Stimulation after Spinal Cord Injury. Journal of neurotrauma. 2018;35(21):2540-53. doi: 10.1089/neu.2017.5584. PubMed PMID: 29786465.
28. Inanici F, Samejima S, Gad P, Edgerton VR, Hofstetter CP, Moritz CT. Transcutaneous Electrical Spinal Stimulation Promotes Long-Term Recovery of Upper Extremity Function in Chronic Tetraplegia. IEEE transactions on neural systems and rehabilitation engineering : a publication of the IEEE Engineering in Medicine and Biology Society. 2018;26(6):1272-8. Epub 2018/06/08. doi: 10.1109/tnsre.2018.2834339. PubMed PMID: 29877852.
29. Rath Mrinal M. Trunk Stability Enabled by Noninvasive Spinal Electrical Stimulation after Spinal Cord Injury. Journal of neurotrauma. 2018;35(21):2540-53.
30. Cohen J. Statistical Power Analysis for the Behavioral Sciences. Second ed. Hillsdale, New Jersey: Lawrence Erlbaum Associates, Publishers; 1988.
31. Sawilowsky S. New Effect Size Rules of Thumb. Journal of Modern Applied Statistics Methods.8(2):467-74.
