# Supplementary material for: Noninvasive spinal stimulation safely enables upright posture in children with spinal cord injury
Source: Nat Commun. 2021 Oct 6;12:5850. doi: 10.1038/s41467-021-26026-z (PMC8494794; doi:10.1038/s41467-021-26026-z)
Supplement: Supplementary file 3 — Reporting Summary [file 41467_2021_26026_MOESM3_ESM.pdf]

nature research

Corresponding author(s): Andreah Behrman

Last updated by author(s): Aug 12, 2021

Reporting Summary

Nature Research wishes to improve the reproducibility of the work that we publish. This form provides structure for consistency and transparency in reporting. For further information on Nature Research policies, see our Editorial Policies and the Editorial Policy Checklist.

Statistics

For all statistical analyses, confirm that the following items are present in the figure legend, table legend, main text, or Methods section.

n/a	Confirmed	
☐	☒	The exact sample size (*n*) for each experimental group/condition, given as a discrete number and unit of measurement
☐	☒	A statement on whether measurements were taken from distinct samples or whether the same sample was measured repeatedly
☐	☒	The statistical test(s) used AND whether they are one- or two-sided<br>*Only common tests should be described solely by name; describe more complex techniques in the Methods section.*
☐	☒	A description of all covariates tested
☐	☒	A description of any assumptions or corrections, such as tests of normality and adjustment for multiple comparisons
☐	☒	A full description of the statistical parameters including central tendency (e.g. means) or other basic estimates (e.g. regression coefficient) AND variation (e.g. standard deviation) or associated estimates of uncertainty (e.g. confidence intervals)
☐	☒	For null hypothesis testing, the test statistic (e.g. *F*, *t*, *r*) with confidence intervals, effect sizes, degrees of freedom and *P* value noted<br>*Give P values as exact values whenever suitable.*
☒	☐	For Bayesian analysis, information on the choice of priors and Markov chain Monte Carlo settings
☒	☐	For hierarchical and complex designs, identification of the appropriate level for tests and full reporting of outcomes
☒	☐	Estimates of effect sizes (e.g. Cohen's *d*, Pearson's *r*), indicating how they were calculated

Our web collection on statistics for biologists contains articles on many of the points above.

Software and code

Policy information about availability of computer code

Data collection	Blood pressure and heart rate monitored with ambulatory blood pressure monitor (ABPM-05,Meditech, Budapest, Hungary) (Figure 1); 3D Kinematics (angular excursions) were measured using MVN BIOMECH Awinda MTW2-3A7G6 sensors (Xsens Technologies B.V. Enschede, Netherlands). The data was sampled at 60Hz and collected in MVN: 2019.2.1 software (XML format). Anteroposterior and mediolateral center of pressure displacements were measured using force plate (Burtec, FP4060-NC-1000). Center of pressure data, sampled at 2000Hz, was acquired in NccHReflex (Labview2017, National instruments) in binary (.bin) format. To synchronize kinematics and center of pressure data a trigger pulse was sent into NccHReflex. The .bin and Xsens files are then converted into text format files (.fns) and Comma Separated (.csv) files using a custom code written in C-sharp (Data Processor 8.9, 2019). Another custom written, C-sharp code (Mvnx2csv 2021.03.09) is then used to combine these two files by sampling up the Xsens data from 60Hz to 2000Hz.
Data analysis	The hemodynamic parameters (blood pressure and heart rate) read outs from the ambulatory blood pressure monitor were manually recorded. The measurements were averaged across the three days at each time point within the experiment in Excel (Microsoft office 365 ProPlus - Excel version 2002). Kinematics and force plate data were exported from the acquisition software as text files and imported into in LabChart 8.1.3 (ADInstruments, USA) where the joint angles and center of pressure displacements were visualized and average peaks and troughs in the angular excursions and center of pressure displacements were calculated for the 10 seconds of stable baseline sitting and sitting with transcutaneous spinal stimulation on using the Data Pad feature. Statistical analysis was performed in SAS 9.4M6 (SAS Inc., Cary, NC).

For manuscripts utilizing custom algorithms or software that are central to the research but not yet described in published literature, software must be made available to editors and reviewers. We strongly encourage code deposition in a community repository (e.g. GitHub). See the Nature Research guidelines for submitting code & software for further information.

Data

Policy information about availability of data

All manuscripts must include a data availability statement. This statement should provide the following information, where applicable:

- Accession codes, unique identifiers, or web links for publicly available datasets
- A list of figures that have associated raw data
- A description of any restrictions on data availability

The hemodynamic outcome measures, incidence of pain, skin redness, autonomic dysreflexia and other safety related outcome measures, as well as trunk kinematic and center of pressure displacement time series data during transcutaneous electrical spinal cord stimulation in children with SCI data generated in this study have been deposited in the Open Data Commons for Spinal Cord Injury (ODC-SCI.org) database and released with the permanent digital object identifier (DOI) numbers as two datasets (DOI#1: 10.34945/F5HP4N, DOI#2: 10.34945/F5NC7X) under a creative commons BY (CC-BY) 4.0 license. These data are publicly available to all registered ODC-SCI users. The access can be obtained by creating an account using institutional email address at ODC-SCI.org. The raw participant demographics-related data are protected and are not available due to data privacy laws. The processed outcome measures data used for generation of figures and tables in the manuscripts are available at ODC-SCI. The data generated in this study are provided in the Supplementary Information/Source Data file. The Human Locomotion Research Center's Volunteer Database is an IRB-approved volunteer database managed through a web-link with public access to volunteer as a potential research candidate/volunteer (https://victoryoverparalysis.org/participate-in-research/). The database is an IRB-approved volunteer database: IRB 06.0647: Development of the KSCIRC Translational Research Database for Potential Research Volunteers. Access to use the database for recruitment of research subjects is not public and not available to the public, but only via institutional IRB approval requested by researchers.

Field-specific reporting

Please select the one below that is the best fit for your research. If you are not sure, read the appropriate sections before making your selection.

☒ Life sciences ☐ Behavioural & social sciences ☐ Ecological, evolutionary & environmental sciences

For a reference copy of the document with all sections, see nature.com/documents/nr-reporting-summary-flat.pdf

Life sciences study design

All studies must disclose on these points even when the disclosure is negative.

Sample size	A sample size of 8 was chosen because it provides a power to detect an effect size of 1.2 on a baseline - stimulation on continuous measure (classified as very large). This sample size was adequate to obtain the outcome variabilities that will help in the statistical planning of a larger study, sufficiently powered to detect a smaller effect size. One subject was excluded from the study due to pain with stimulation. The final results are reported on n =7.
Data exclusions	Trunk muscle electromyography (EMG) data was collected during the study, however, the EMG signal was saturated with the stimulation artifact due to the proximity of the anode to the recording electrodes. This data was therefore excluded from the analysis.
Replication	The experimental procedures for application of transcutaneous spinal cord stimulation were replicated 3 times on 3 separate days (once per day, 3 days) for n=7. For n=1 the initial application of stimulation caused intolerable pain due to the presence of allodynia at the site of stimulation and thus this participant was excluded from further experiments. Within each experiment hemodynamic stability and self-reported pain outcomes (using FACES or VAS scores) were measured at 3 pre-specified time points within the experiment: baseline (no stimulation), with stimulation on at three stimulation locations and at the end of experiments (with no stimulation). Additional blood pressure or heart rate measures were performed if the participant reported or showed signs of autonomic dysreflexia. Stimulation was turned on and ramped up to the upright-posture inducing thresholds at T11, then L1, then C5. For each stimulation site one trial was performed each day. The stimulation trials at any given location were replicated only if the initial stimulation parameters did not lead to induction of upright posture. In one case stimulation at higher intensities did not evoke strong enough trunk extension response. After the parameters were adjusted in that particular case (switch of frequency from 15 Hz to 30Hz for L1 stimulation site), the robust trunk extension was obtained in that participant. Stimulation with the parameters used in the current experiment was relatively less effective for a participant with neuromuscular scoliosis.
Randomization	This study is a within subjects, repeated measures design with all subjects serving as their own control. Therefore randomization was not performed.
Blinding	There was no blinding in the current study. Participants were informed when the stimulation was on and could inform us if there was any sensory response or discomfort. The majority of the participants, however, did not feel the stimulation at the lumbosacral sites below the injury and were oblivious to its presence during testing.<br>To address the proof-of-principal for stimulation to enable upright trunk posture, trunk extension was compared between stimulation on and stimulation off. The authors acknowledge that the unblinded data analysis of the trunk control outcomes is an oversight in analysis to contribute to unbiased reporting. We are, however, transparent with access to the data and believe that mitigates potential biases that could have influenced our results or conclusions. We are also providing a source data file with the full time-series data (continuous recording of trunk angles from baseline, stimulation ramp up and stimulation ramp down) for all the participants, along with stimulation intensities that were used to analyze the data and generate Figure 3 as described in the methods. The data is also publicly available on ODC-SCI.org as described in data availability. This affords independent/unbiased replication of our analysis.

Reporting for specific materials, systems and methods

We require information from authors about some types of materials, experimental systems and methods used in many studies. Here, indicate whether each material, system or method listed is relevant to your study. If you are not sure if a list item applies to your research, read the appropriate section before selecting a response.

Materials & experimental systems

n/a	Involved in the study
x	Antibodies
x	Eukaryotic cell lines
x	Palaeontology and archaeology
x	Animals and other organisms
	x Human research participants
	x Clinical data
x	Dual use research of concern

Methods

n/a	Involved in the study
x	ChIP-seq
x	Flow cytometry
x	MRI-based neuroimaging

Human research participants

Policy information about studies involving human research participants

Population characteristics	Eight participants were enrolled, ages 2-15 years who met the following inclusion criteria: history of chronic, acquired upper motor neuron SCI (traumatic or non-traumatic); discharged from in-patient rehabilitation, trunk control deficit as either documented with the Segmental Assessment of Trunk Control (SATCo, score < 20) or reported/observed inability to sit fully upright and without use of arm support or difficulty reaching while maintaining posture. The sex of the participants and other demographic-based co-variates are excluded from reporting due to Editorial policies and the concern for a potential re-identification of the participants when more than two indirect identifiers are present.
Recruitment	The research manager conducted an inquiry of the Human Locomotion Research Center's Volunteer Database (UofL Study IRB #06.0647) for potentially eligible candidates based on the study eligibility criteria. Participants were screened first, over the phone or in person with parent/caregiver, via an informed consent process by the principal investigator to review the study purpose, procedures, risks, and eligibility criteria. As potential candidates may have entered their information in the database potentially months and possibly years ago, eligibility was reviewed specific to this study's criteria and current candidate status. Candidates passing the first screen and interested in participating, underwent a second, live medical screen conducted by the study physician using the study eligibility to validate a candidate's eligibility. All participants who underwent the screen by the study physician met eligibility criteria and were afforded the opportunity to participate. Candidates willing to participate signed informed consent (parent/guardian) with assent by children ≥7 years. Travel distance to the study site (and the presence of covid restrictions) may have precluded study screening and participation, but would have no impact on study results.
Ethics oversight	The University of Louisville Institutional Review Board (IRB) approved this study (IRB protocol #19.0377)

Note that full information on the approval of the study protocol must also be provided in the manuscript.

Clinical data

Policy information about clinical studies

All manuscripts should comply with the ICMJEguidelines for publication of clinical research and a completedCONSORT checklist must be included with all submissions.

Clinical trial registration	NCT03975634
Study protocol	The trial is registered on clinicaltrials.gov.
Data collection	Clinical site: Frazier Rehab and Neuroscience Institute is a 15-story rehabilitation facility that provides in- and out-patient healthcare services including for individuals with spinal cord injury. (SCI). The 'Discovery Cove' – Pediatric NeuroRecovery Research Lab where the data collection took place is located on the 6th floor of Frazier Rehab Institute and was specially designed for conducting research in children with neurological dysfunction. The Pediatric Neurorecovery Program is part of the Kentucky Spinal Cord Injury Research Center which is a nationally- and internationally-recognized research center targeting repair of the spinal cord with basic science research and a translational research program focusing on recovery, health, and quality of life for individuals with spinal cord injury (SCI). Recruitment and screening for the eligible participants began in 06/19, with the first participant enrolled in 08/19. The last (8th) participant was enrolled in 01/20. Further enrollment of the participants was halted due to COVID-19 pandemic.
Outcomes	Predefined primary outcomes:<br>1. Incidence of skin irritation<br>Skin color, particularly change in skin color to pink indicating irritation in the location of the stimulating electrode placement will be assessed prior to stimulation experiments and immediately after; incidence of pink or redness or irritation<br>2a Faces Pain Scale-Revised (scale 0-10)<br>Faces Pain Scale - Revised is a self-report measure of pain intensity developed for children (C.L. Hicks et al. Pain 93 (2001). It will be used to score the sensation of pain on 0 (min - no pain)-to-10 (max - worst pain ever) metric. The faces scale will be presented to the

participant (ages 3-8) prior to the experiment for baseline measurement, during stimulation and following the experiment.
2b. Visual Analog Scale (0-10)
To assess pain in the participants ages 8 and above, Visual Analog Scale (self-reported measure) will be presented with 0 corresponding to no pain and 10 corresponding to the "worst pain ever"; the scale will be presented at baseline measurement, during stimulation and following the experiment.
3. Hemodynamic parameters
Brachial arm blood pressure (mmHg) and heart rate (beats per minute) measured at baseline, during stimulation, end of experiments and if a child presents with symptoms of autonomic dysreflexia (e.g. facial flushing, reporting of headache, etc)
4. Angular excursions of trunk during stimulation optimization
Trunk kinematics (degrees of flexion/extension) in cervical, thoracic and lumbar regions;
Predefined secondary outcomes:
1. Compliance rate
Compliance - number of sessions missed and reason, willingness to continue participation.
2. Center of pressure displacement during trunk control assessment
The distance (mm) of the center of pressure displacement will be measured in mediolateral; anterior -posterior directions as the stimulation intensity is increased
